# Unraveling wheat’s response to salt stress during early growth stages through transcriptomic analysis and co-expression network profiling

**DOI:** 10.1186/s12863-024-01221-1

**Published:** 2024-04-12

**Authors:** Wei Wang, Sufang Huang, Zhi Wang, Pingping Cao, Meng Luo, Fengzhi Wang

**Affiliations:** 1 Cangzhou Academy of Agriculture and Forestry Sciences, 061001 Cangzhou, Hebei China; 2 Hebei Key Laboratory of Drought-Alkali Tolerance in Wheat, 061001 Cangzhou, Hebei China; 3 Shanghai Majorbio Bio-pharm Technology Co., Ltd, 200120 Shanghai, China

**Keywords:** Wheat, Salt stress, RNAseq, Co-expression network

## Abstract

**Background:**

Soil salinization is one of the vital factors threatening the world’s food security. To reveal the biological mechanism of response to salt stress in wheat, this study was conducted to resolve the transcription level difference to salt stress between CM6005 (salt-tolerant) and KN9204 (salt-sensitive) at the germination and seedling stage.

**Results:**

To investigate the molecular mechanism underlying salt tolerance in wheat, we conducted comprehensive transcriptome analyses at the seedling and germination stages. Two wheat cultivars, CM6005 (salt-tolerant) and KN9204 (salt-sensitive) were subjected to salt treatment, resulting in a total of 24 transcriptomes. Through expression-network analysis, we identified 17 modules, 16 and 13 of which highly correlate with salt tolerance-related phenotypes in the germination and seedling stages, respectively. Moreover, we identified candidate Hub genes associated with specific modules and explored their regulatory relationships using co-expression data. Enrichment analysis revealed specific enrichment of gibberellin-related terms and pathways in CM6005, highlighting the potential importance of gibberellin regulation in enhancing salt tolerance. In contrast, KN9204 exhibited specific enrichment in glutathione-related terms and activities, suggesting the involvement of glutathione-mediated antioxidant mechanisms in conferring resistance to salt stress. Additionally, glucose transport was found to be a fundamental mechanism for salt tolerance during wheat seedling and germination stages, indicating its potential universality in wheat. Wheat plants improve their resilience and productivity by utilizing adaptive mechanisms like adjusting osmotic balance, bolstering antioxidant defenses, accumulating compatible solutes, altering root morphology, and regulating hormones, enabling them to better withstand extended periods of salt stress.

**Conclusion:**

Through utilizing transcriptome-level analysis employing WGCNA, we have revealed a potential regulatory mechanism that governs the response to salt stress and recovery in wheat cultivars. Furthermore, we have identified key candidate central genes that play a crucial role in this mechanism. These central genes are likely to be vital components within the gene expression network associated with salt tolerance. The findings of this study strongly support the molecular breeding of salt-tolerant wheat, particularly by utilizing the genetic advancements based on CM6005 and KN9204.

**Supplementary Information:**

The online version contains supplementary material available at 10.1186/s12863-024-01221-1.

## Introduction

Wheat (*Triticum aestivum L.*) is a significant staple crop, and its production significantly impacts global food security. The global extent of saline soil covers approximately 9.54 million hectares [1]. With the shortage of water resources and the increasing severity of soil salinization, wheat production faces severe drought and salt stresses, which restricts grain yields. Soil salinization can cause severe infiltration rib, persecution, and ionized toxicity to plants. Salt stress is usually caused by the high neutral salt content such as NaCl and Na_2_SO_4_, and NaCl is the leading cause of natural saline lands. On the one hand, the high salt concentration of soil causes serious infiltration ribs to plants, resulting in difficulties in root water absorption and leaf water loss, and subsequently the decline of cell turgor pressure, and death of cells, tissues, and even the whole plants [2]. Large amounts of Na^+^ destroy the ion balance inside plants, causing ion toxicity to plants. Excessive Na^+^ also destroys the plant’s photosynthetic system, inhibits root growth, reduces biomass, and finally causes leaf drying [3, 4]. It is found that the number of tillers and spikelets and the weight of straw and grain were all drastically reduced in compact saline treatment [5]. Therefore, breeding for salt-tolerant wheat cultivars has great significance in guaranteeing food security in front of soil salinization.

Although we have made progress in understanding the factors determining wheat salt tolerance, the genetic basis is still unclear. Previous studies have identified many quantitative trait loci (QTLs) for salt tolerance [6–8]. Some reported QTLs on chromosomes 3B, 5B, and 5D were found to co-localize with the QTLs detected in a recent study indicating the stable effects of these loci in salt tolerance [9–12]. However, genetic studies based on QTLs are often limited by genetic backgrounds and low resolution of gene detection. In addition, salt tolerance is an extremely complex biological trait and is genetically regulated by multiple genes associated with ion transport, osmotic regulation, antioxidation, and signaling transduction [13–16]. Salt tolerance breeding requires a holistic understanding of gene regulatory networks and salt tolerance phenotypes in crops. High-throughput transcriptome analysis provides an effective insight into the pathways related to salt tolerance and their underlying mechanisms.

Wheat grain yield is significantly limited by soil salinity, a critical constraint that intensifies due to factors like global climate change, seawater intrusion, and urbanization [17]. Recently, studies on plant resistance have combined transcriptome and metabolome profiling. Wheat has investigated responses to salt stress and phosphorus deficiency using transcriptomic profiling [17–21]. However, some studies have used transcriptomic and metabolomic profiling techniques to investigate the mechanisms underlying wheat’s ability to withstand salt stress. Recently, some studies combined transcriptome and metabolome analyses to investigate how plants react to abiotic stress [18–20].

In this study, we used Kenong9204 (KN9204) and Cangmai6005 (CM6005) to study salt tolerance, of which KN9204 has a diverse genetic basis, high yield potential, and high nitrogen use efficiency. It is widely used as a promising parent in wheat breeding [22, 23]. Although KN9204 is widely used as a promising parent in breeding, it is sensitive to salt tolerance. CM6005 has better environmental adaption such as drought resistance, salt tolerance, and stable yield. This study aims at mining salt tolerance-related gene regulatory networks by comparing differences between the two wheat cultivars in transcription levels. The results of this study will help to reveal the functional regulation of salt tolerance of CM6005 and provide the genetic basis and potential valuable loci for wheat improvement.

## Materials and methods

### Variety source and phenotype mensuration

Two wheat cultivars were sourced: the salt-resistant variety CM6005 from the Cangzhou Academy of Agriculture and Forestry Sciences (CAAFS) and the salt-sensitive KN9204 from the Hebei Key Laboratory of Crop Salt-Alkali Tolerance Evaluation and Genetic Improvement, China. The study was carried out in CAAFS’s artificial climate chamber. Two types of soils were used for the experiment: a control soil with a 0.1% salt concentration and a treated soil with a 0.5% salt concentration. Before potting, these soils were aerated, crushed, and uniformly mixed. The soils were then placed into pots measuring 18 cm in length, 11 cm in width, and 6 cm in height, with each pot containing 400 g of soil. Table 1 provides details about the physical and chemical characteristics of these experimental soils. Each treatment was replicated three times, as detailed in Table S1.

**Table 1:** Physicochemical properties of the test soil

Index	control soil(0.1%)	salt soil(0.5%)
total salt content(g.kg^− 1^)	1.1210 ± 0.0283	4.4940 ± 0.7947
pH	8.2060 ± 0.0456	8.1280 ± 0.0700
CO_3_^2−^+HCO_3_^−^(g.kg^− 1^)	0.2180 ± 0.0116	0.1660 ± 0.0169
Na^+^(g.kg^− 1^)	0.1880 ± 0.0058	1.2580 ± 0.1421
Cl^+^(g.kg^− 1^)	0.0820 ± 0.0116	1.3580 ± 0.1020
Mg^2+^(g.kg^− 1^)	0.0220 ± 0.0073	0.1360 ± 0.0614
K^+^(g.kg^− 1^)	0.0072 ± 0.0024	0.0087 ± 0.0010
Ca^2+^(g.kg^− 1^)	0.0718 ± 0.0035	0.1625 ± 0.0394
SO_4_^2−^(g.kg^− 1^)	0.5120 ± 0.0185	1.3660 ± 0.2991
SiO_4_^4−^(g.kg^− 1^)	0.0320 ± 0.0020	0.0280 ± 0.0020

Leaves were collected for RNA-seq analysis at two-time intervals, 7 and 14 days after treatment (dat), corresponding to the germination stage and the seedling stage, respectively. At the 7-day mark, seven parameters associated with salt tolerance were evaluated, including the germination rate, seedling length, number of roots, length of the main root, fresh weight of the shoot, fresh weight of the root, and root-crown ratio. At the 14-day mark, eight different parameters were assessed, such as the growth in height, growth in root length, fresh and dry weights of the above-ground part, as well as the fresh and dry weights of the underground part.

### Biochemical analysis

All the biochemical indices within the leaves were measured using commercial enzyme-linked immunosorbent assay (ELISA- Table S9) kits, following the instructions provided by the manufacturer (MEIMIAN, Jiangsu, China).

### Sequencing sample preparation and mRNA library construction

Leaf-derived total RNA from both control and experimental sets were meticulously isolated employing the TRIzol reagent (Invitrogen, Carlsbad, CA, USA). This was followed by a rigorous DNase I treatment (QIAGEN, Shanghai, China) to obliterate any contaminating genomic DNA. The integrity of the RNA, indicative of degradation and contamination, was assiduously ascertained on 1% agarose gels. Thereafter, the RNA’s fidelity was gauged via the 2100 Bioanalyzer (Agilent,), and its concentration was quantified utilizing the ND-2000 (NanoDrop Technologies Inc., Wilmington, DE, USA). An aliquot of 1 µg RNA from each specimen was earmarked as the input material. Only RNA specimens of impeccable quality (as characterized by OD260/280 values between 1.8 and 2.2, OD260/230 values equal to or above 2.0, RIN values equal to or above 6.5, and a 28 S:18 S ratio equal to or above 1.0) were harnessed for the generation of sequencing libraries. Transcriptome libraries for RNA-seq were meticulously constructed by the protocols delineated in the TruSeq™ RNA Sample Preparation Kit from Illumina (San Diego, CA, USA). In the initial phase, messenger RNA (mRNA) was diligently isolated employing the polyA selection approach, facilitated by oligo (CM6005) beads, followed by targeted fragmentation utilizing a specialized fragmentation buffer. Subsequently, double-stranded complementary DNA (cDNA) was synthesized by harnessing the capabilities of the SuperScript Double-Stranded cDNA Synthesis Kit (Invitrogen, city, CA, USA), in conjunction with random hexamer primers. The synthesized cDNA was then adeptly subjected to a series of intricate molecular procedures, including end-repair, phosphorylation, and the addition of an adenine (‘A’) base, all scrupulously executed in alignment with Illumina’s proprietary library construction guidelines. The libraries were further refined through a meticulous size-selection process, targeting cDNA fragments of approximately 300 bp in length, achieved through the use of 2% Low Range Ultra Agarose. This was succeeded by a PCR amplification phase, deploying Phusion DNA polymerase (NEB, Typically used at a concentration of 0.025 to 0.075 units/µl), extending through 15 precise PCR cycles. Following rigorous quantification via the TBS380 instrument, the final paired-end RNA-seq libraries were prepared for sequencing, culminating in a sophisticated sequencing run on the Illumina NovaSeq 6000 sequencer, achieving an in-depth read (length of 2 × 150 bp).

### Identification of different expression genes and function enrichment analysis

The initial paired-end raw reads were systematically trimmed and subjected to rigorous quality control employing the fastp tool (Version 0.19.5, https://github.com/OpenGene/fastp) [24], utilizing the default parameters. Subsequently, the resulting high-quality, clean reads were independently aligned to the reference genome, with precise orientation, utilizing the HISAT2 software (Version 2.1.0, http://ccb.jhu.edu/software/hisat2/index.shtml) [25]. Transcripts’ expression levels were comprehensively computed by the transcripts per million reads (TPM) metric, and RSEM software (Version 1.3.1, http://deweylab.biostat.wisc.edu/rsem/) [26] was used to quantify gene abundances.

Simultaneously, raw data were subjected to an initial processing phase through the employment of SeqPrep1 and Sickle2, utilizing default parameters. Following the methodical trimming of adapter sequences, excision of low-quality bases, and filtration of short reads, the remaining clean reads were independently aligned to the reference genome of *Triticum aestivum* (Triticum_aestivum:https://plants.ensembl.org/Triticum_aestivum/Info/Index), adhering to the orientation mode with HISAT2 software. The alignment criteria were stringently defined: sequencing reads must uniquely correspond to the genome, with an allowance for up to 2 mismatches, while precluding any insertions or deletions. Concurrently, the clean data were scrutinized for Q20, Q30, GC-content, and sequence duplication levels. These refined, high-integrity reads were then earmarked for successive analyses. Finally, each transcript’s gene expression level was meticulously calculated by the fragments per kilobase of the exon model per million mapped reads (FPKM) protocol, an approach that takes into account both the read count mapped to the given transcript and its length.

To identify differentially expressed genes (DEGs) under various experimental conditions, specifically comparisons between CM6005 vs. KN9204 and control vs. salt treatment, we first constructed a statistical model based on observed gene counts. Leveraging the Bayes theorem, we ascertained the *p*-value signifying the intergroup disparity for each gene, utilizing the sophisticated DESeq2 package (an empirical framework for digital gene expression analysis in R) [27]. Genes meeting the rigorous criteria of a *p*-value (FDR) less than 0.05 and an absolute fold change (FC) of at least 2 were classified as DEGs. After this, an in-depth Gene Ontology (GO) and Kyoto Encyclopedia of Genes and Genomes (KEGG) pathway enrichment assessment of the identified DEGs was executed using the advanced ClusterProfiler software package [28].

### Utilizing co-expression network analysis to establish modules

To delve deeper into the identification of DEGs associated with salt tolerance-associated phenotypes, we employed the advanced analytical capabilities of the WGCNA software package to architect co-expression networks. By rigorously assessing both the independence and the mean connectivity degrees across distinct modules with varying power values, we ascertained the optimal power value. Modules were discerned using the intrinsic network construction function of the WGCNA software package [29], leveraging default parameters. Genes exhibiting maximal intra-modular connectivity are categorically designated as hub genes. To achieve a nuanced understanding of the architecture, we pinpointed the paramount 10 hub genes for every module, harnessing the analytical prowess of ClusterONE within the Cytoscape software framework [30, 31]. This methodical approach underpins a sophisticated analysis, elucidating the intricate dynamics and pivotal genes governing salt tolerance in the studied organisms.

### qRT‑PCR analysis

To validate our findings, we meticulously selected ten prominently expressed hub genes for quantitative real-time PCR (qRT-PCR) analysis. Total RNA isolated from wheat specimens was subsequently reverse-transcribed to yield the first-strand cDNA using a premier reverse transcription kit optimized for real-time PCR (TaKaRa). The requisite primers, synthesized by the reputable Majorbio (Shanghai, China), are delineated in Supplementary Table S2 (Additional file 1). qRT-PCR assays were adeptly executed employing the state-of-the-art Step One qTOWER 2.0/2.2 Quantitative Real-Time PCR Thermal Cyclers from Analytik Jena, Germany. Further refining our procedure, we utilized the SCILOGEX D3024R High-Speed Refrigerated Micro-Centrifuge from the USA, Scandrop100, and pipettes with volumes of 10µL, 100µL, and 1000µL sourced from Bio-rad, To ensure homogeneity, we centrifuged the reaction mixtures at 6,000 rpm for a duration of 1 min, allowing all components to settle at the bottom of the tubes. Our qRT-PCR thermal profile encompassed an initial denaturation at 95 °C for 3 min, followed by 39 amplification cycles of 95 °C for 10 s and 60 °C for 30 s. Post-amplification, melt curve analysis was performed, incrementally raising the temperature from 60 to 95 °C with a ramp rate of + 1 °C per cycle, while sustaining each temperature for 4 s. we conducted three technical replicates for the qRT-PCR analysis.

For quantifying relative gene expression, one particular sample was designated as the reference, with the housekeeping gene GAPDH employed as an internal normalization control. Utilizing the 2^−△△^Ct methodology [32], relative gene expression values were deduced based on the dataset from a single biological replicate for every treatment (as detailed in Additional file 2). Notably, the advanced qPCRsoft3.2 software (2.0.0.3/ https://www.bio-rad.com/en-us/category/real-time-pcr-systems?ID=059db09c-88a4-44ad-99f8-78635d8d54db) provides an automated avenue for the computation of relative gene expression.

## Results

### Phenotypic differences between CM6005 and KN9204 in the salt-treated condition

**Fig. 1:**
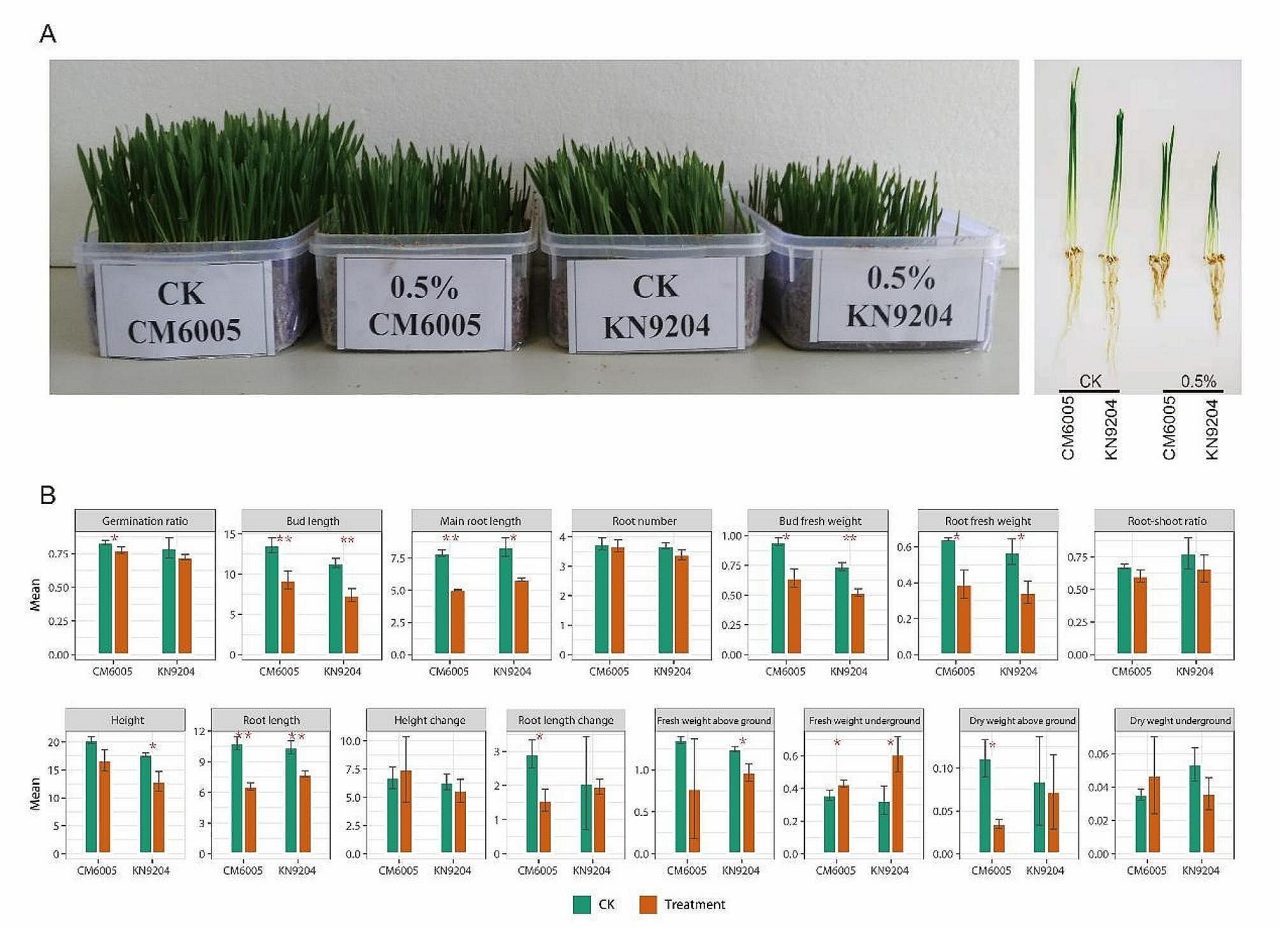
Phenotypic features of CM6005 and KN9204 under different conditions. **A** Potting situation of CM6005 and KN9204 under CK and salt stress. **B** Phenotypic difference of CM6005 and KN9204 between CK and salt stress (the data units refers to the units of measurement used for the observed traits: millimeters, centimeters, etc)

Under the salt-treated (0.5% total salt content) condition, CM6005 showed stronger growth potential than KN9204. However, the root development of CM6005 appeared to be more sensitive to salt treatment (Fig. 1A). To further dissect the phenotypic characteristics of different varieties under treatment, we detected multiple phenotypes related to salt tolerance (Fig. 1B). By comparing phenotypic changes seedling length, main root length, seedling fresh weight, root fresh weight, root length, and fresh weight underground were significantly different between the salt treatment and the control, with an increase in fresh weight underground and a decrease in other parameters under the salt treatment. This result illustrates the consistent effect of the salt treatment on both varieties. Notably, the percentage of germination, root length, and dry weight above ground significantly reduced under the salt-treatment condition only in CM6005. The height and fresh weight of the above-ground part were significantly reduced under the salt-treatment condition only in KN9204, which illustrates the specificity effect of the salt treatment on different varieties.

### Differences in the transcript level between control and salt treatment at 7 and 14 d

**Fig. 2:**
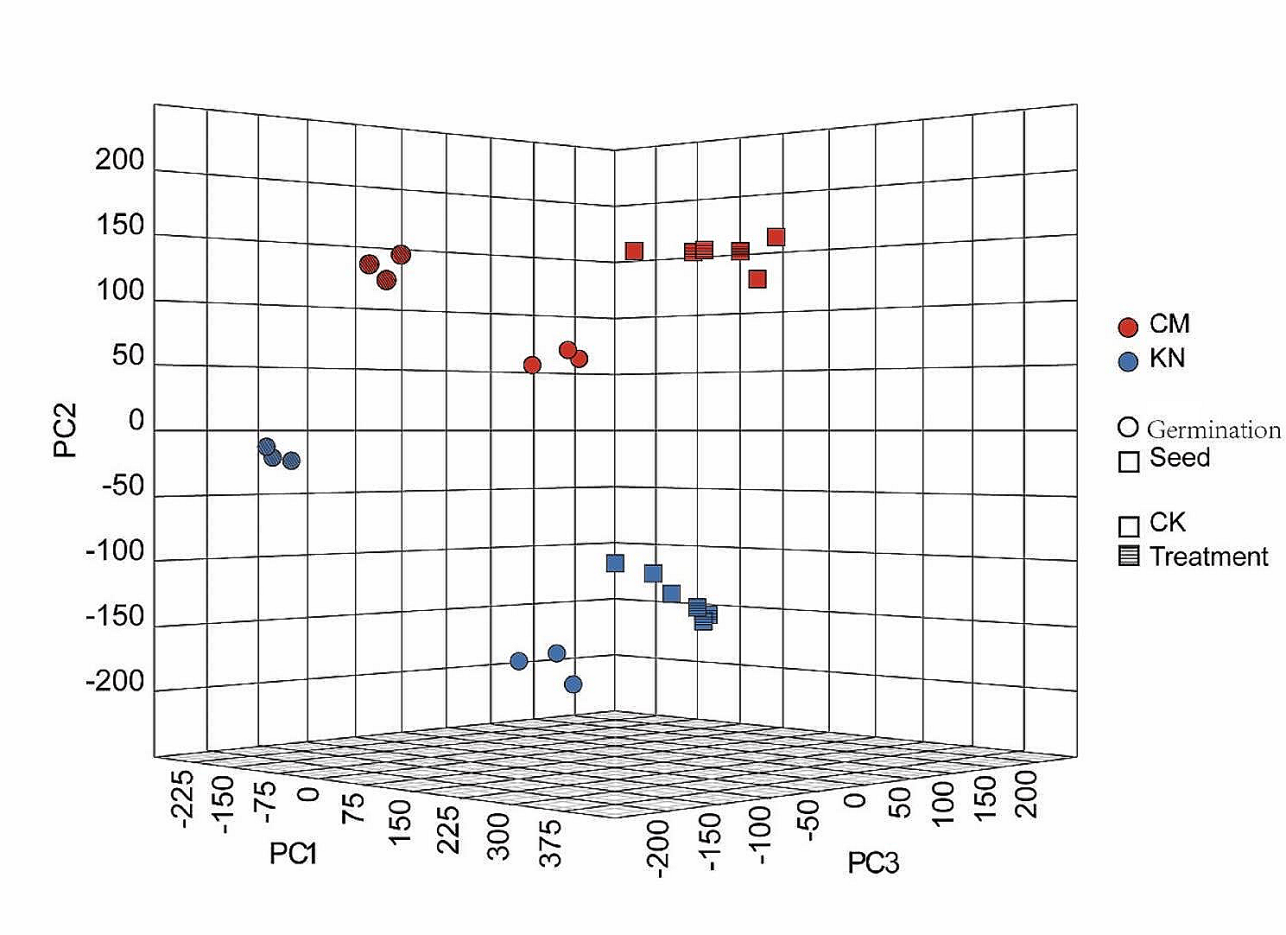
Three-dimensional plot of principal component analysis for wide genomic gene expression

**Fig. 3:**
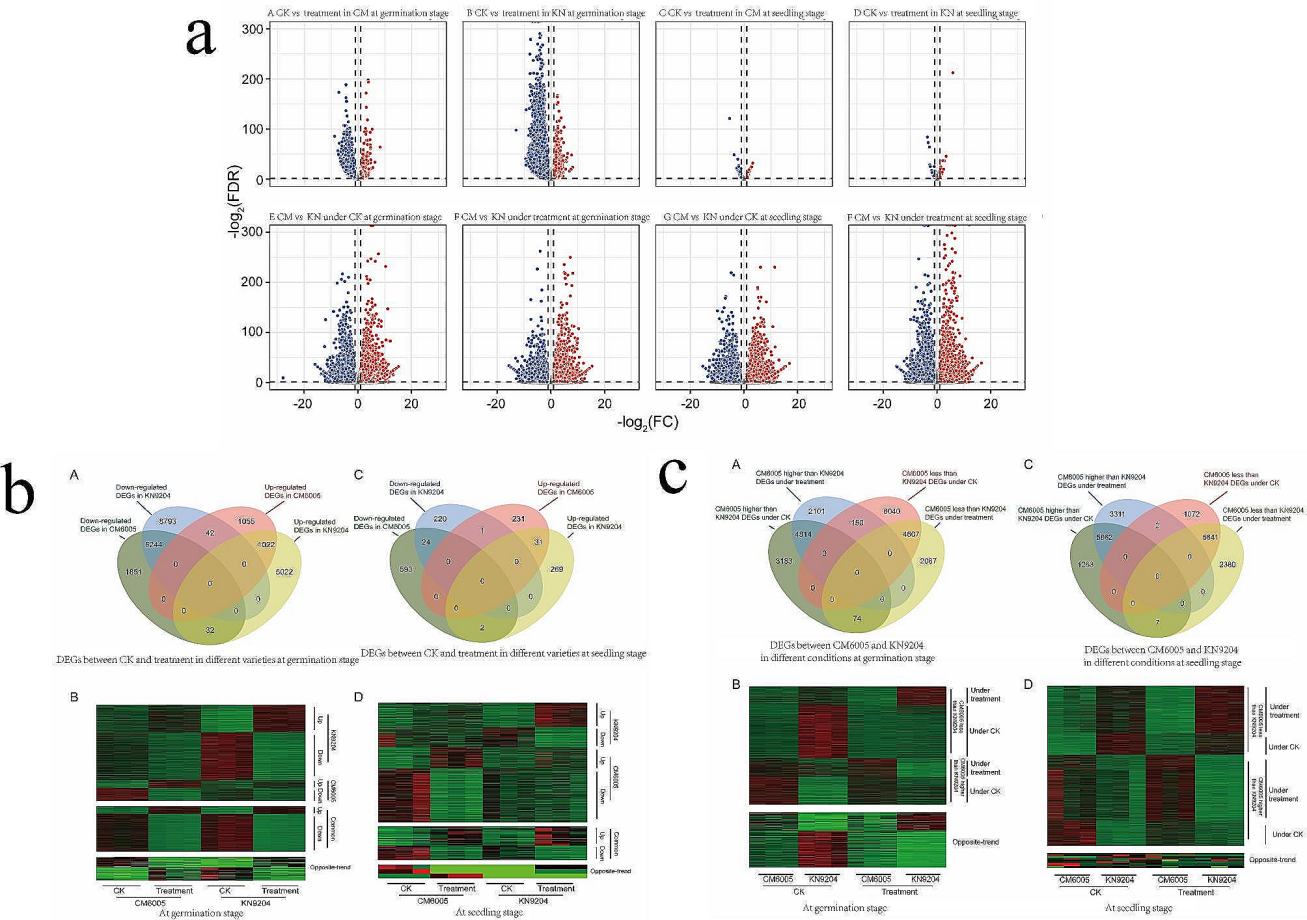
Different expression genes under different conditions. (**a**) Blue points represented significantly downregulated genes and red points represented significantly upregulated genes. CM: CM6005; KN: KN9204; (**b**) Comparison of different expression genes (DEGs) between CK and treatment at germination and seedling stage. **A** At the germination stage the number of DEGs between CK and treatment. **B** At germination stage expression heatmap of DEGs between CK and treatment. **C** At the seedling stage the number of DEGs between CK and treatment. **D** At seedling stage expression heatmap of DEGs between CK and treatment; (**c**) Comparison of different expression genes (DEGs) between CM6005 and KN9204 at germination and seedling stage. **A** At the germination stage the number of DEGs between CM6005 and KN9204. **B** At germination stage expression heatmap of DEGs between CM6005 and KN9204. **C** At the seedling stage the number of DEGs between CM6005 and KN9204. **D** At seedling stage expression heatmap of DEGs between CM6005 and KN9204

Owing to early salt tolerance in wheat being affected by seedling and germination stage, we separately determined the transcriptomes of CM6005 and KN9204 under control and salt treatment to reveal the biological basis of differences related to salt tolerance between the two varieties. We extracted the first three principal components through principal component analysis (PCA) to distinguish the difference between sample groups. The corresponding results showed that CM6005 vs. KN9024, germination stage vs. seedling stage, and control vs. salt treatment were markedly distinguished (Fig. 2, Table S3), which indicated that global transcriptional levels of sample groups had marked differences and samples within the same group had excellent repeatability. We detected DEGs between the control and the salt treatment in CM6005 and KN9204 (Fig. 3, Table S4). Whether CM6005 or KN9204, the number of DEGs at the germination stage was markedly more than that of the seedling stage, which indicates that the germination stage is more sensitive to salt stress. These DEGs were divided into three categories, variety-specific DEGs, common DEGs, and opposite-trend DEGs. At the germination stage, there were 1851 genes specifically down-regulated and 1055 genes specifically up-regulated in CM6005 under the salt-treated condition (Fig. 3, Table S4). There were 8793 genes specifically down-regulated and 5022 genes specifically up-regulated in KN9204 under the salt-treated condition. Meanwhile, 5244 genes were significantly down-regulated in both CM6005 and KN9204, and 1022 genes were significantly up-regulated in both varieties. Furthermore, 74 DEGs showed opposite trends in both varieties, where 42 genes were significantly down-regulated in KN9204 and up-regulated in CM6005, and 32 genes were significantly down-regulated in CM6005 and up-regulated in KN9204.

At the seedling stage, there were 593 genes specifically down-regulated and 231 up-regulated in CM6005 under the salt-treated condition (Fig. 3, Table S4). Meanwhile, 220 genes were specifically down-regulated and 269 genes were specifically up-regulated in KN9204 under the salt-treated condition. Twenty-four genes were significantly down-regulated and 31 genes were significantly up-regulated in both varieties. Furthermore, 3 DEGs showed opposite trends in both varieties.

### Differences in the transcript levels between CM6005 and KN9204

The difference in salt tolerance between CM6005 and KN9204 was reflected not only in DEGs between control and salt treatment but also in the relative change of DEGs between the two cultivars under different conditions. One of the situations was that DEGs associated with the difference in salt tolerance between CM6005 and KN9204 could be detected under only one condition, either control or salt treatment, which can be defined as DEGs based on relative changes. Another situation was that DEGs between CM6005 and KN9204 could be detected under both control and salt treatment, but the direction of DEGs changed was reversed, which can be defined as DEGs with opposite trends. Therefore, we detected DEGs between CM6005 and KN9204 under different conditions (Fig. 3, Table S4). The DEGs associated with salt tolerance at the germination stage were more than those at the seeding stage, which also indicates that the germination stage was more sensitive to salt stress. At the seedling stage, DEGs between control and salt treatment were less than DEGs based on relative changes, which indicates that the relative changes of DEGs between CM6005 and KN9204 under different conditions were the major factor related to salt tolerance.

At the germination stage, the expression levels of 3183 genes in CM6005 were significantly higher than those in KN9204 under the control condition, but there was no significant difference under salt treatment. Contrarily, the expression levels of 2101 genes in CM6005 were significantly higher than those in KN9204 under the salt-treated condition, but there was no significant difference under the control condition. Similarly, there were 6040 genes under the control condition with lower expression in CM6005 than those in KN9204 but were not significant under the salt-treated condition. Correspondingly, there were 2087 genes with lower expression in CM6005 than those in KN9204 under the salt-treated condition but were not significant under the control condition. Moreover, the expression levels of 150 genes in CM6005 were significantly higher than those in KN9204 under the salt-treated condition. Under the control condition, their expression levels were significantly lower than those in KN9204. Contrarily, the expression levels of 74 genes in CM6005 were significantly lower than those in KN9204 under the salt-treated condition but significantly higher than those in KN9204 under the control condition.

At the seedling stage, there were expression levels of 1263 genes in CM6005 were significantly higher than those in KN9204 under the control condition, but there was no significant difference under salt treatment. In addition, there were expression levels of 3311 genes in CM6005 were significantly higher than those in KN9204 under the salt-treated condition, but there was no significant difference under the control condition. Similarly, there were 1072 genes under the control condition with lower expression in CM6005 than those in KN9204 and not significant under the salt-treated condition. Under the salt-treated condition, there were 2380 genes with lower expression in CM6005 than in those in KN9204, but not significantly under the control condition. Furthermore, the expression levels of two genes in CM6005 were significantly higher than those in KN9204 under the salt-treated condition but significantly lower under the control condition. In contrast, the expression levels of 7 genes in CM6005 were significantly lower than those in KN9204 under the salt-treated condition but significantly higher than KN9204 under the control condition.

### Function categories and pathways related to salt tolerance at germination and seedling stages

Based on the different analyses of the transcriptional level, we divided all DEGs into five categories: common DEGs, specific DEGs to CM6005, specific DEGs to KN9204, DEGs based on relative changes, and DEGs with opposite trends. These DEGs reflected the biological basis of salt tolerance in different viewpoints, where common DEGs reflected consistent features of salt tolerance in CM6005 and KN9204 and the other DEGs reflected specific features in one of the varieties.

**Fig. 4:**
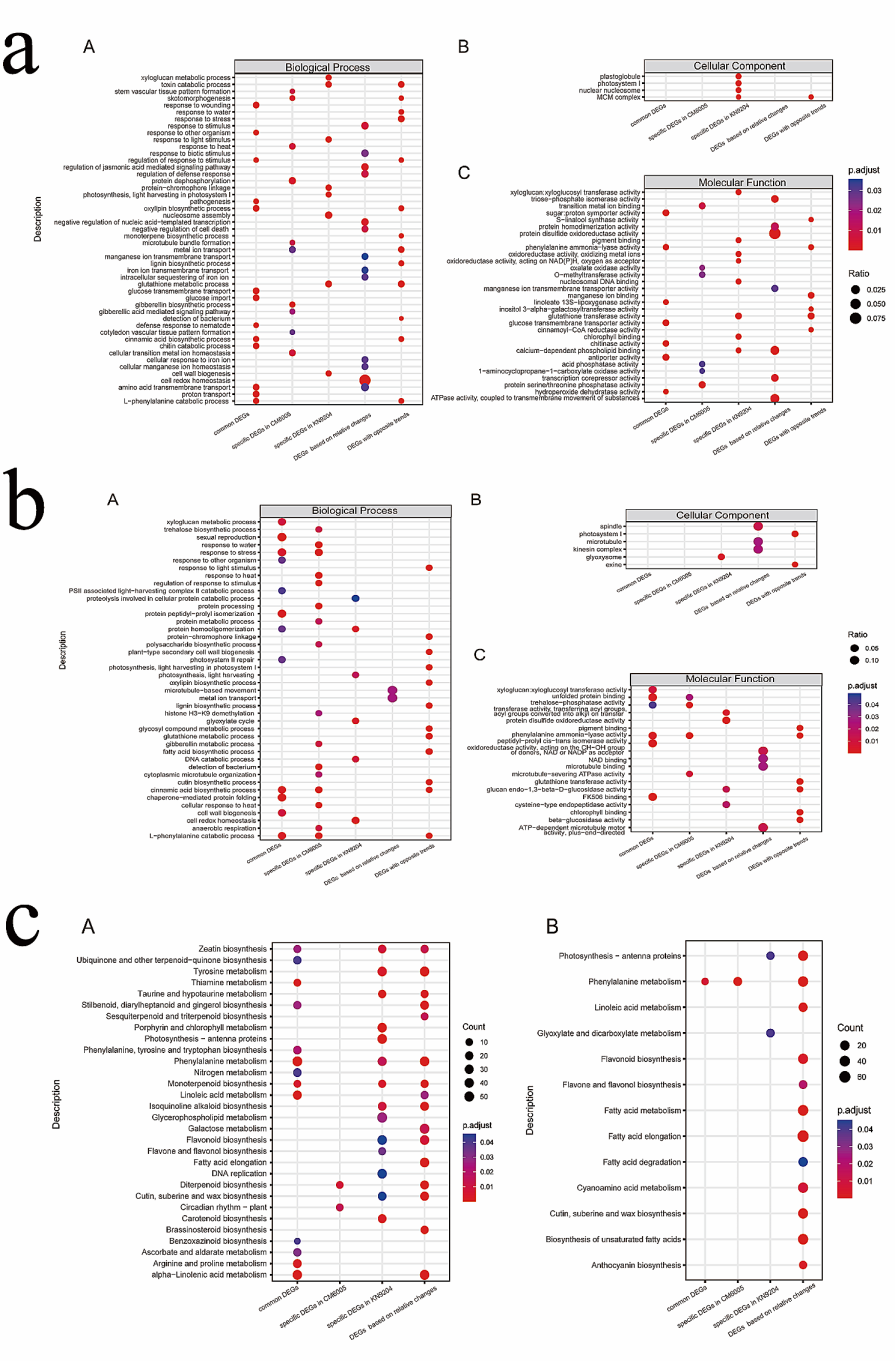
(**a**) Comparison of biological process (**A**), cellular component (**B**), and molecular function (**C**) terms among different type DEGs at the germination stage; (**b**) Comparison of biological process (**A**), cellular component (**B**), and molecular function (**C**) terms among different DEGs at the seedling stage; (**c**) Comparison of KEGG pathway among different types DEGs at germination (**A**) and seedling (**B**) stage

To reveal the functional category and pathway related to salt tolerance at the germination and seedling stages, we performed an enrichment analysis of DEGs (Fig. 4, Table S5). At the germination stage, these DEGs were mainly related to biological processes, followed by molecular functions. Only the specific DEGs in KN9204 and the DGEs with opposite trends were related to the cellular component. Based on the common DEGs, we enriched oxylipin biosynthetic process (GO:0031408), glucose transmembrane transport (GO:1,904,659), higher hydroperoxide dehydratase activity (GO:0047987), response to wounding (GO:0009611), and amino acid transmembrane transport (GO:0003333), which indicated that these terms were common features of CM6005 and KN9024 under salt treatment. The salt tolerance of CM6005 was specifically associated with skotomorphogenesis (GO:0009647), response to heat (GO:0009408), and stem vascular tissue pattern formation (GO:0010222). However, the salt tolerance of KN9204 was specifically associated with the toxin catabolic process (GO:0009407), response to light stimulus (GO:0009416), and xyloglucan metabolic process (GO:0010411). Distinctly, the terms enriched by specific DEGs had a marked difference between CM6005 and KN9024. According to the DEGs based on relative changes and those with opposite trends, other terms were detected, such as response to stress (GO:0006950), protein disulfide oxidoreductase activity (GO:0015035), etc., by which functional categories associated with salt tolerance were ambiguity.

**Fig. 5:**
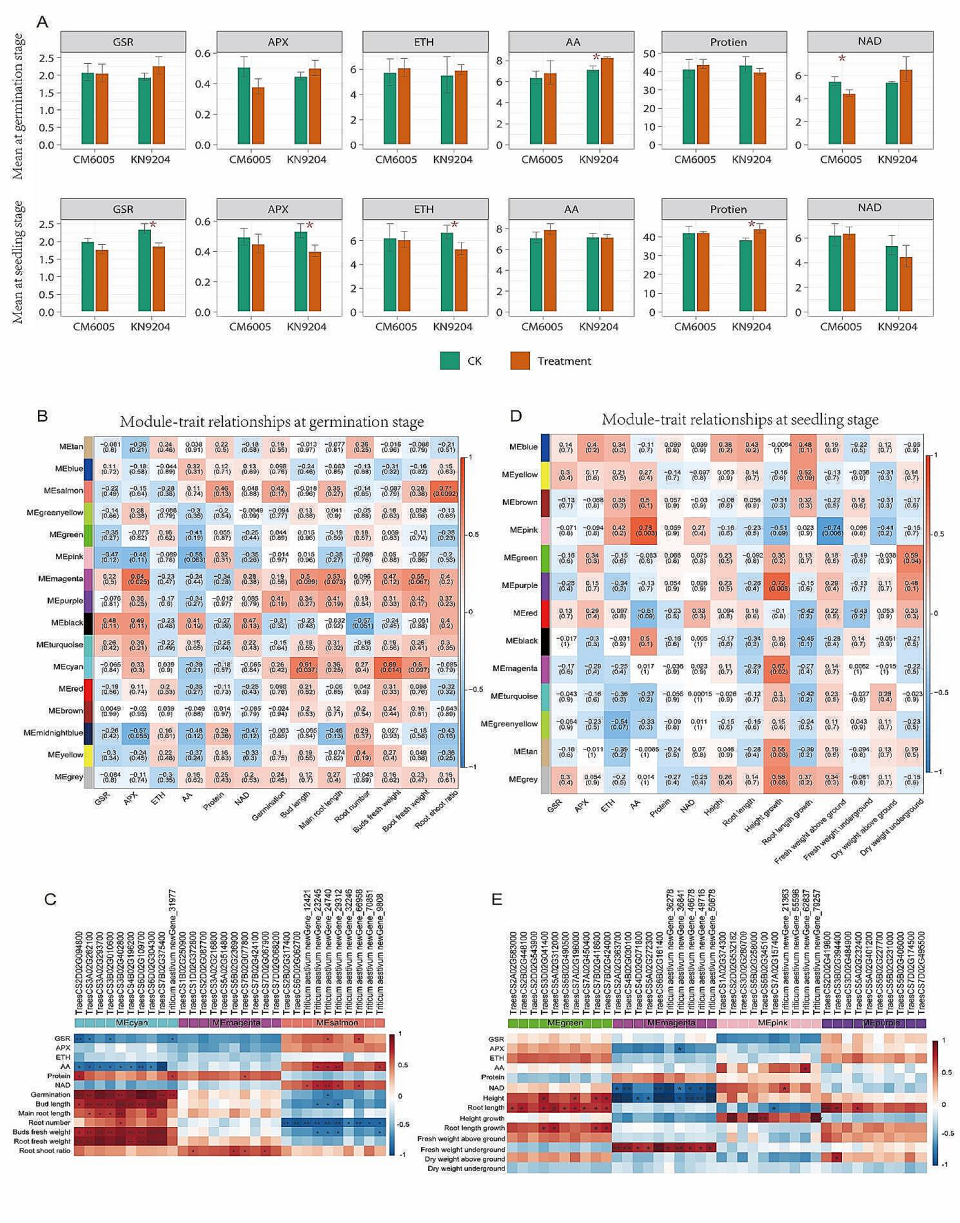
Weighted correlation network analysis for different expression genes. **A** Difference of metabolons related to salt tolerance at germination and seedling stage. **B** The correlation between phenotypes and gene modules at the germination stage. **C** The correlation between phenotypes and hub genes at the germination stage. **D** The correlation between phenotypes and gene modules at the seedling stage. **E** The correlation between phenotypes and hub genes at the seedling stage. *, *P* < 0.05; **, *P* < 0.001; ***, *P* < 0.0001

At the seedling stage, the terms enriched were different from those at the germination stage, and only some of the terms were repeated (Fig. 4, Table S5), for example, such as response to wounding (GO:0009611), response to heat (GO:0009408), response to water (GO:0009415), and stress response (GO:0006950). Based on the common DEGs, we enriched cell wall biogenesis (GO:0051260), photosystem II repair (GO:0010206), and trehalose-phosphatase activity(GO:0004805). The salt tolerance of CM6005 was specifically associated with the regulation of response to stimulus (GO:0009061), gibberellin metabolic process (GO:0009685), and polysaccharide biosynthetic process. However, the salt tolerance of KN9204 was specifically associated with protein disulfide oxidoreductase activity (GO:0015035), cell redox homeostasis (GO:0045454), and glyoxysome (GO:0009514). According to the DEGs based on relative changes and those with opposite trends, other terms associated with salt tolerance at the seedling stage were detected, including fatty acid biosynthetic process (GO:0006633), photosystem I (GO:0009522), and glutathione metabolic process (GO:0006749). These results indicated that the salt tolerance at the germination and seedling stages was associated with not only different biological terms but also common terms. These different biological terms indicated the differences in salt tolerance across the germination and seedling stages. While commons terms revealed a more general biological basis of salt tolerance.

We identified function pathways associated with salt tolerance (Fig. 4, Table S6). At the germination stage, phenylalanine metabolism (ko00360), alpha-Linolenic acid metabolism (ko00592), and arginine and proline metabolism (ko00330) were commonly detected as biological pathways associated with salt tolerance. The circadian rhythm (ko04712) was a specific functional pathway of CM6005 at the germination stage. The photosynthesis-antenna proteins (ko00196), porphyrin and chlorophyll metabolism (ko00860), carotenoid biosynthesis (ko00906), glycerophospholipid metabolism (ko00564), and flavone and flavonol biosynthesis (ko00944) were specific functional pathways of KN9024 at the germination stage. At the seedling stage, the functional pathways enriched were less than those at the germination stage. Most pathways were detected by the DEGs based on relative changes. Phenylalanine metabolism (ko00360) was repeatedly detected by multiple types of DEGs. Glyoxylate and dicarboxylate metabolism (ko00630) was the specific functional pathway of KN9024. These results further illustrated that the germination stage was more important than the seedling stage for salt tolerance.

### Screening of co-expression modules associated with salt tolerance

To further reveal the relationship between DEGs and salt tolerance, we filtered six important metabolons related to salt tolerance, including GSR, APX, ETH, AA, NAD, and total protein. At the germination stage, AA and NAD showed a significant difference between the control and the salt treatment in KN9204 and CM6005, respectively. At the seedling stage, GSR, APX, ETH, and total protein showed a significant difference between control and salt treatment in KN9204 (Fig. 9A, Table S6).

Based on the expression levels of common DEGs, specific DEGs in CM6005, specific DEGs in KN9204, DEGs based on relative changes, and DEGs with opposite trends, we constructed a correlation matrix and identified software threshold for germination and seedling stages. Using hierarchical clustering, we divided the DEGs into multiple co-expression modules, of which the expression patterns were different. Further, we calculated Pearson correlation coefficients between modules and phenotypes to identify modules associated with salt tolerance. At the germination stage, 16 co-expression modules were identified (Fig. 9B), where MEmagenta, MEcyan, and MEsalmon modules were significantly associated with salt tolerance phenotypes. MEmagenta was associated with GSR (*r* = 0.64, *P* = 0.025). MEcyanwas associated with germination length and germination fresh weight (*r* = 0.61, *P* = 0.037 and *r* = 0.69, *P* = 0.014, respectively). MEsalmon was associated with a root-shoot ratio (*r* = 0.71, *P* = 0.009). These results indicate that the DEGs in these modules influence salt tolerance by acting on these corresponding phenotypes.

At the seedling stage, 13 co-expression modules were identified (Fig. 9D, Table S6). MEpink, MEgreen, MEpurple, and MEmagenta(seedling) were significantly associated with phenotypes related to salt tolerance. MEplink was associated with AA and dry weight above ground (*r* = 0.78, *P* = 0.003 and *r* = -0.74, *P* = 0.006, respectively). MEgreen was associated with dry weight underground (*r* = 0.59, *P* = 0.04). MEpurple and MEmagenta (seedling) were associated with height growth (*r* = 0.72, *P* = 0.008 and *r* = 0.67, *P* = 0.002, respectively).

### Identification of hub genes associated with salt tolerance

To excavate hub genes, we constructed a co-expression network for each associated module. Moreover, we calculated the connectivity degree of each gene in the modules, where the top ten genes with the highest connectivity degrees were considered to be hub genes. At the germination stage, 30 hub genes associated with GSR, root/shoot ratio, germination length, and germination fresh weight were found. At the seedling stage, 40 hub genes were associated with AA, dry weight above ground, dry weight underground, and height growth. Then, we detected the association between hub genes with phenotypes related to salt tolerance at different stages. At the germination stage, the hub genes were mainly associated with AA, germination, germination length, root length, shoot fresh weight, and root fresh weight (Fig. 9C, Table S7). At the seedling stage, the hub genes were mainly associated with NAD, height, root length, height growth, root length growth, and fresh weight underground (Fig. 9E, Table S7). Because the hub genes had high connectivity degrees, changes in hub genes were closely related to variations of many other genes.

### Validation of the hub genes by RT-qPCR

In CM6005 and KN9024, we selected 12 genes for qRT-PCR verification (Figure S1-2), and the primer information is shown in Table S2. The results suggested the high reliability of the RNA-seq data. To verify the reproducibility and authenticity of the RNA-seq data, 12 hub genes with high expression in key modules were selected for qRT-PCR analysis (Table S2). As shown in Fig. 4, Table S6, qRT-PCR results of all 12 genes were consistent with the expression pattern of RNA-seq data. Genes significantly up-regulated in RNA-Seq data also exhibited an up-regulation in qPCR, and vice versa. These results also confirmed the reliability of the RNA-Seq data.

## Discussion

Salt tolerance is a complex and closely related property to the environment, influencing many physiological processes of crops. We measured multiple traits and metabolites associated with salt tolerance in wheat cultivars CM6005 and KN9024 contrasting in the responses to salt stress. Through quantitative results of RNA-seq, we identified DEGs under different conditions and detected several hub genes associated with salt tolerance phenotypes. This study reveals gene regulatory networks for salt tolerance and provides the basis for molecular breeding of salt tolerance in wheat.

We selected CM6005 and KN9204 with typical tolerance and sensitivity to salt, respectively, which showed a significant difference in several salt tolerance-related traits. Through comparing genome-wide transcription levels between CM6005 and KN9204 at the germination and seedling stages, we found that the number of DEGs at the germination stage was more than that at the seedling stage. This indicates that the germination stage was the most important period of salt tolerance. This result is consistent with the previous studies [33]. Salt tolerance at the germination stage is reflected in the water absorption and expansion capacity of seeds, which is to resist osmotic stress [34]. While the main salt tolerance mechanism at the seedling stage is the resistance to high concentrations of ions.

We identified five types of salt tolerance related DEGs including common DEGs, specific DEGs in CM6005, specific DEGs in KN9204, DEGs based on relative changes and DEGs with opposite trends, by comparing CM6005 vs. KN9204, and control vs. salt treatment. The common DEGs reflected the same responses to salt stress in CM6005 and KN9204, which may explain the general mechanism of salt tolerance in wheat. For instance, amino acid transmembrane transport (GO:0003333) was enriched in common DEGs at the germination stage and associated with multiple abiotic stresses including salt stress in different plants [35–38]. The terms glucose transmembrane transport (GO:1,904,659), glucose import (GO:0046323), and glucose transmembrane transporter activity (GO:0046323) were enriched only in common DEGs at the germination stage. This indicates the importance of glucose transport as a fundamental mechanism of salt tolerance in wheat at the germination stage. Moreover, in some horticultural crops, glucose transport was closely related to multiple stresses including cold, heat, salt, and drought [39–42]. When subjected to stresses, plants could maintain cell turgor pressure through glucose transport to improve resistance and normal growth. Glucose transport was the universal mechanism of plant resistance. In addition, higher hydroperoxide dehydratase activity (GO:0047987) could accelerate the decomposition of reactive oxygen, maintain the integrity of the cell membrane and attenuate the destruction of chloroplasts by salt stress, which is an important mechanism of plant stress resistance [43–46]. The result of KEGG enrichment analysis for common DEGs at the germination stage shows that thiamine metabolism (ko00730) is a common metabolism pathway associated with salt tolerance in wheat. As an important coenzyme, thiamine plays multiple roles in various metabolic activities of cells and also in responding to environmental stress stimuli. Thiamine can improve the oxidation state of mitochondria in plant cells and enhance the activity of pyruvate dehydrogenase. It is also useful for cells to release reactive oxygen species quickly when they are stimulated by stress factors, activate downstream signals, and induce plant resistance [47–49]. The pathways, phenylalanine, tyrosine, and tryptophan biosynthesis (ko00400), nitrogen metabolism (ko00910), and arginine and proline metabolism (ko00330), were related to the nitrogen cycle. The relationship between nitrogen metabolism and plant resistance to stress is complex, mainly from nitrogen absorption, assimilation, and amino acid metabolism involved in plant stress resistance. The resistance of plants to abiotic stress is influenced by various physiological mechanisms, such as the regulation of ion balance, stabilization of cell morphology and protein structure, maintenance of hormone balance and cellular metabolic levels, reduction of reactive oxygen species generation, and enhancement of chlorophyll synthesis. With the increase in salt concentration, the sodium and chloridion ingested by plants were increased, resulting in a decrease in the ratio of potassium to sodium ions. The accumulation of chloride in the plant would stimulate ethylene synthesis, promote leaf shedding, and inhibit plant growth [50–52]. Several studies showed that nitrogen application under salt stress could reduce the uptake and accumulation of potassium and sodium ions, and increase the ratio of potassium to sodium ions in plants [53–57]. However, the common DEGs at the seedling stage were different from those at the germination stage, and the response to salt tolerance was not sensitive.

The specific DEGs in CM6005 or KN9204 reflected the variety-specific salt tolerance mechanisms. The enriched terms and pathways related to salt tolerance were not overlapped between CM6005 and KN9204. The enriched terms and pathways in CM6005 could have a greater effect on salt stress. The terms gibberellin biosynthetic process (GO:0009686), gibberellic acid mediated signaling pathway (GO:0009740), and gibberellin metabolic process (GO:0009685) were specifically enriched in CM6005 at the germination and seedling stages. As an important plant hormone, gibberellin not only regulates seed germination, leaf extension, and elongation of stem and root but also participates in the process of tolerating many abiotic stresses in plants. Under salt stress, plants could improve salt tolerance by reducing gibberellin [13, 58–60]. This result indicates that the regulation of gibberellin may be one of the important mechanisms of higher salt tolerance of CM6005, and genetic improvement in gibberellin regulation should be considered in salt-tolerant breeding. In KN9204, specific-enriched terms, glutathione metabolic process (GO:0006749) and glutathione transferase activity (GO:0004364) were related to abiotic stresses. The glutathione could be able to exert resistance to salt stress through antopxidationmediated by repairing membrane phospholipid damage and inhibiting microsomal peroxidation reaction [61–64]. Considering the sensitivity to salt stress of KN9204, the salt resistance mechanism based on glutathione did not appear to have a sufficiently positive effect. Xyloglucan could enhance salt tolerance by regulating the stomatal closure to prevent excessive water loss [65, 66]. Interestingly, xyloglucan-related terms were KN9204-specific at the germination stage under salt stress, but at the seedling stage, it was enriched by common DEGs, which illustrates that salt tolerance mechanisms can be transformed at different stages, and its importance varies at different stages. The two DEG types, DEGs based on relative changes and DEGs with opposite trends, reflect the relative difference in gene expression under salt stress. These DEGs could better reflect the corresponding characteristics to salt stress in wheat, especially DEGs with opposite trends. At the germination stage, resistance to stress-related terms, such as response to stress (GO:0006950), regulation of response to stimulus (GO:0048583), and response to stimulus (GO:0050896), were enriched by these DEGs. However, at the seedling stage, these terms were enriched only by specific DEGs in CM6005, which could be a cause of salt tolerance in CM6005. Therefore, improving the stress response at the seedling stage may be important in salt tolerance breeding. Moreover, CM6005 carries these genetic factors corresponding to seedling adversity.

By assessing the correlation between gene expression and their form, we categorized differentially expressed genes (DEGs) into distinct modules. Within these modules, we identified hub genes that played a crucial role in salt tolerance, thereby pinpointing key genes associated with this trait. At the germination stage, the hub genes were mainly associated with AA, germination, germination length, root length, germination fresh weight, and root fresh weight. TraesCS2D02G094800 is a member of the bHLH transcription factor family and regulates the development of lateral roots [67], which is very important at the germination stage in wheat. More importantly, genes in this family regulate multiple abiotic stresses including low temperature, drought, and salt stresses in other plants [68–71]. TraesCS3A02G262100 is homologous to AtLRK10L1.2, which is involved in ABA-mediated signaling and drought resistance in Arabidopsis thaliana [72]. TraesCS3A02G293700 is a bZIP transcription factor. As one of the largest regulatory families of transcription factors in the plant, it plays an important role in physiological processes such as abiotic stress response and development. This transcription factor can be induced by drought, salt, cold, and abscisic acid, and interacts with cis-acting elements in the promoter region of stress-related genes to regulate the transcript level of target genes, and then regulates plant tolerance [73–75]. TraesCS6D02G304300 is homologous to ROC8 in rice (*Oryza sativa* L.), and ROC8 can regulate the direction of leaf curl by affecting the formation and development [76]. While in wheat it could be related to the development of germination. TraesCS7B02G077800 is a member of the GRAS domain family and homologous to Scarecrow-like protein 9 in Arabidopsis. This protein is involved in the development of roots and stems. In addition, TaSCL14, the gene in the same family of wheat, is highly expressed in stems and roots and is a regulator of antioxidative stress [77]. TraesCS1D02G372800 is a member of the mitogen-activated protein kinase (MAPK) family. This family is closely associated with abiotic stress. RiceOsMAPK33 maintains plant body homeostasis and enhances tolerance to high salt by regulating sodium/potassium ions [78]. At the seedling stage, the hub genes were mainly associated with NAD, height, root length, height growth, root length growth, and fresh weight underground. TraesCS5B02G490500 belongs to the Zinc finger protein transcript factor and is homologous to ZAT1 in Arabidopsis. Rice OsZFP182 can activate the expression of genes, such as OsP5CS and OsLEA3, and promote the accumulation of osmoregulatory substances such as proline and soluble sugars under stress conditions [79]. TraesCS4B02G030100 codes calcium-dependent protein kinase (CDPK), which is an important calcium ion receptor. Biological and abiotic stresses cause elevated cytosolic calcium ion levels. CDPK can discriminate calcium single and act on downstream interacting proteins, causing a range of physiological responses. In rice, overexpression of OsCDPK7/13/21 increased tolerance to salt, drought, and cold stress [80–83]. Similarly, TraesCS3D02G041400 codes probable calmodulin-like protein (CML) and is homologous to OsCML16, which is associated with rice response to low-temperature adversity [84]. In wheat, it can be related to the development of seedlings under salt stress. TraesCS7B02G418600 is a member of the WRKY transcription factor family, which plays an important role in plant response to abiotic stresses, and expression can be induced by different abiotic stresses [85, 86].TaWRKY46, a gene in the same family, can enhance osmotic stress tolerance by regulating the transduction of the ABA signaling pathway [87]. TraesCS2B02G448100 encodes an ethylene-responsive transcription factor (ERF), which is an important regulator in the salt stress signaling response pathway. Heterologous expression of barley (*Hordeum vulgares* L.) *HvDREB1* in transgenic Arabidopsis enhances tolerance to high salt stress [88]. These findings show that hub genes change between the germination and seedling stages. In addition, we predict that some new genes are related to salt tolerance, some of which may play a core role as hub genes. These hub genes need to be further resolved and their functions remain to be investigated.

## Conclusions

We conducted a comprehensive analysis of transcript-level differences in response to salt stress between CM6005 and KN9204 at the germination and seedling stages. Our investigation revealed that CM6005 exhibits a significant advantage in salt tolerance, particularly during the germination stage. By carefully categorizing differentially expressed genes (DEGs), we examined the overall gene networks associated with salt tolerance, as well as the specific gene networks unique to CM6005 and KN9204. This in-depth analysis allowed us to uncover potential mechanisms underlying the divergent salt tolerance observed between CM6005 and KN9204. Furthermore, we identified hub genes that serve as central components within the gene networks related to salt tolerance. These findings not only enhance our understanding of the molecular basis of salt tolerance in wheat but also have significant implications for molecular breeding programs aimed at developing salt-tolerant wheat varieties. By utilizing the knowledge gained from this study, researchers and breeders can make informed decisions and apply targeted approaches to improve the salt tolerance of wheat through genetic advancements and breeding strategies. Ultimately, our findings contribute to the overall goal of ensuring sustainable wheat production and global food security in the face of increasing soil salinity challenges.

### Electronic supplementary material

Below is the link to the electronic supplementary material.

Supplementary Material 1

Supplementary Material 2

Supplementary Material 3

Supplementary Material 4

Supplementary Material 5

Supplementary Material 6

Supplementary Material 7

Supplementary Material 8

Supplementary Material 9

Supplementary Material 10

Supplementary Material 11

Supplementary Material 12

## Data Availability

All datasets generated or assessed during this research are encompassed within the manuscript and its supplementary files. The datasets pertinent to this investigation can be accessed in the National Center for Biotechnology Information (NCBI) BioProject repository with the accession identifier PRJNA906318.
