# Supplementary material for: Unraveling wheat’s response to salt stress during early growth stages through transcriptomic analysis and co-expression network profiling
Source: BMC Genom Data. 2024 Apr 12;25:36. doi: 10.1186/s12863-024-01221-1 (PMC11015659; doi:10.1186/s12863-024-01221-1)
Supplement: Supplementary file 11 — Supplementary Material 11 [file 12863_2024_1221_MOESM11_ESM.docx]

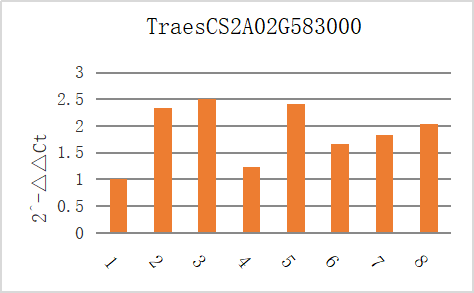

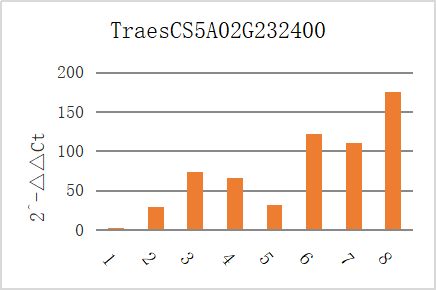

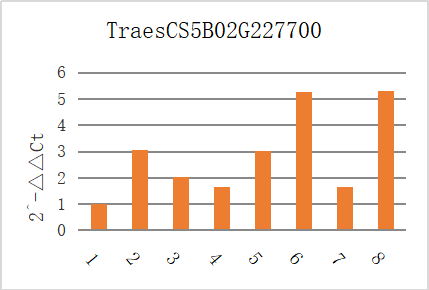

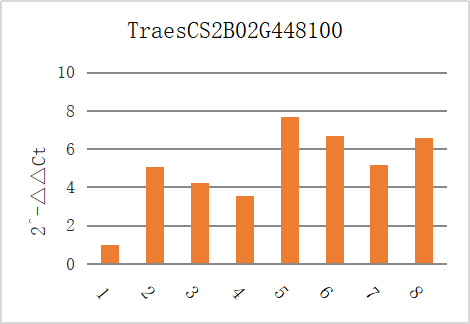

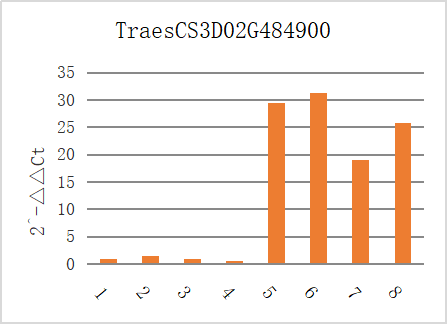

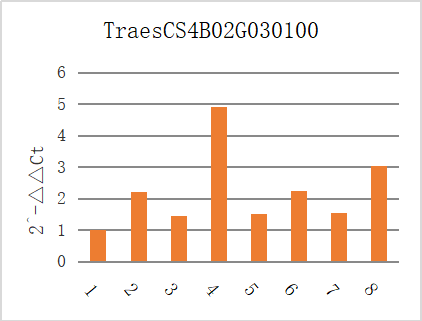


**Fig. S1** The relative expression levels of 6 genes in seeding stage. We only choose one sample from the experimental sampling code (Table S1-1-8)


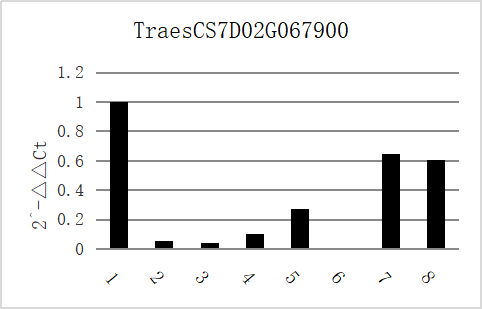

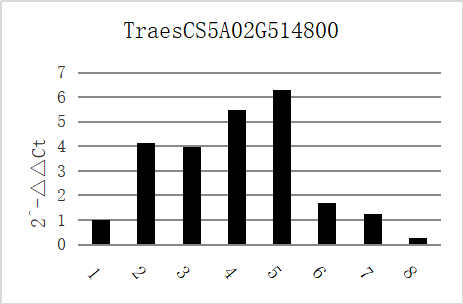

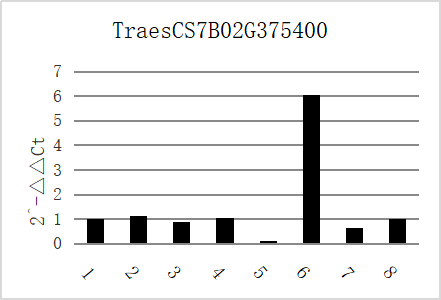

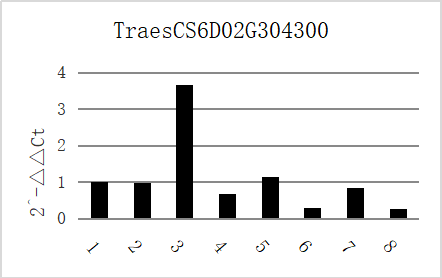

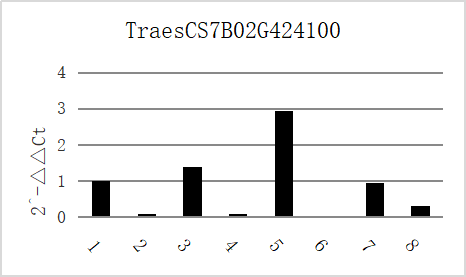

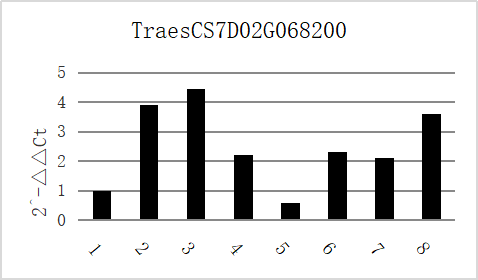


**Fig. S2** The relative expression levels of 6 genes in germination stage. We only choose one sample from the experimental sampling code (Table S1-1-8)
