# Supplementary material for: Unraveling wheat’s response to salt stress during early growth stages through transcriptomic analysis and co-expression network profiling
Source: BMC Genom Data. 2024 Apr 12;25:36. doi: 10.1186/s12863-024-01221-1 (PMC11015659; doi:10.1186/s12863-024-01221-1)
Supplement: Supplementary file 12 — Supplementary Material 12 [file 12863_2024_1221_MOESM12_ESM.docx]

R codes for PCA and factor analysis

# Install and load the necessary libraries

install.packages(c("psych", "ggplot2"))

library(psych)

library(ggplot2)

phenotype_data<-read.csv("yaqi.csv",header = T)

phenotype_data<-read.csv("miaoqi.csv",header = T)

#PCA

pca_result <- prcomp(phenotype_data, scale = TRUE)

write.csv(pca_result$x,file = "pca.csv",quote = F,row.names = F)

#PCA result

sum<-summary(pca_result)

sum$importance

write.csv(sum$importance,file = "Cumu.csv",quote = F,row.names = T)

# factor analysis

factor_analysis_result <- factanal(phenotype_data ,factors = 3, rotation = "varimax")

#factor loading

write.csv(factor_analysis_result$loadings,file = "factor.csv",quote = F,row.names = T)

print(factor_analysis_result$loadings)

# Plot the cumulative variance contribution of PCA

cumulative_variance <- cumsum(pca_result$sdev^2) / sum(pca_result$sdev^2)

pdf("pca.pdf",width = 5,height = 5)

plot(1:length(cumulative_variance), cumulative_variance, type = "b",

xlab = "Number of Principal Components", ylab = "Cumulative Variance")

dev.off()

The results of phenotypic traits of PCA and factor analysis at the germination and seedling stage

Seedling stage:

Table S1 Summary OF PCA

|  | PC1 | PC2 | PC3 | PC4 | PC5 | PC6 | PC7 | PC8 |
| --- | --- | --- | --- | --- | --- | --- | --- | --- |
| Standard deviation | 1.909 | 1.296 | 1.019 | 0.904 | 0.732 | 0.482 | 0.183 | 0.138 |
| Proportion of Variance | 0.455 | 0.210 | 0.130 | 0.102 | 0.067 | 0.029 | 0.004 | 0.002 |
| Cumulative Proportion | 0.455 | 0.665 | 0.795 | 0.897 | 0.964 | 0.993 | 0.998 | 1.000 |


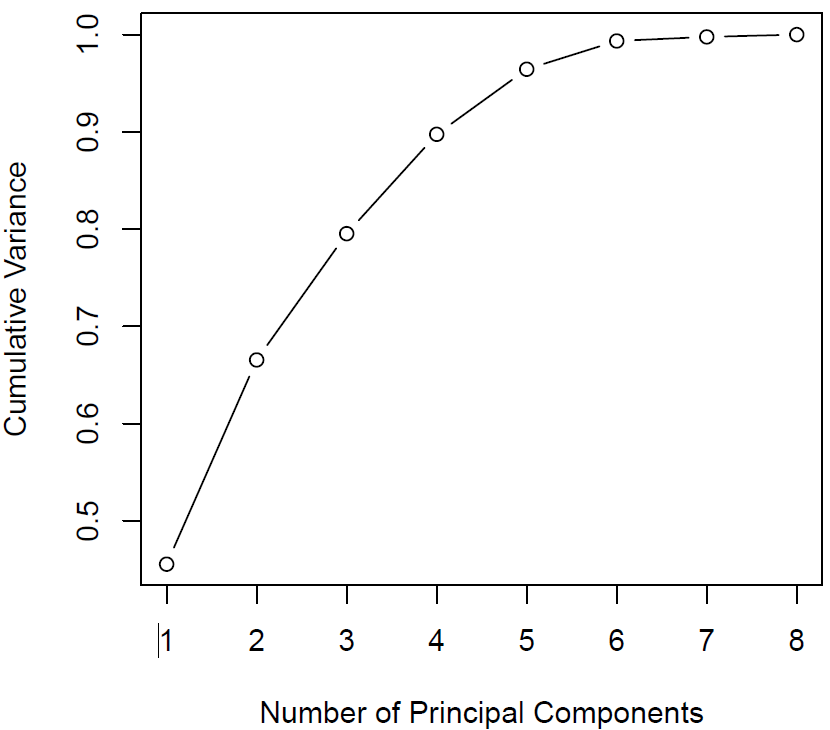


Table S2 Summary OF factor analysis

|  | Factor1 | Factor2 | Factor3 |
| --- | --- | --- | --- |
| Plant Height | 0.476 | 0.661 | 0.435 |
| Root Length | 0.915 | 0.379 | -0.116 |
| Increase in Plant Height | 0.012 | 0.144 | 0.987 |
| Increase in Root Length | 0.759 | -0.074 | 0.024 |
| Aboveground Fresh Weight | 0.759 | 0.193 | 0.471 |
| Belowground Fresh Weight | -0.201 | -0.960 | -0.183 |
| Aboveground Dry Weight | 0.641 | -0.056 | 0.086 |
| Belowground Dry Weight | -0.258 | 0.394 | -0.015 |

Germination:

Table S3 Summary OF PCA

|  | PC1 | PC2 | PC3 | PC4 | PC5 | PC6 | PC7 |
| --- | --- | --- | --- | --- | --- | --- | --- |
| Standard deviation | 2.116 | 1.220 | 0.756 | 0.581 | 0.331 | 0.118 | 0.026 |
| Proportion of Variance | 0.640 | 0.213 | 0.082 | 0.048 | 0.016 | 0.002 | 0.000 |
| Cumulative Proportion | 0.640 | 0.852 | 0.934 | 0.982 | 0.998 | 1.000 | 1.000 |


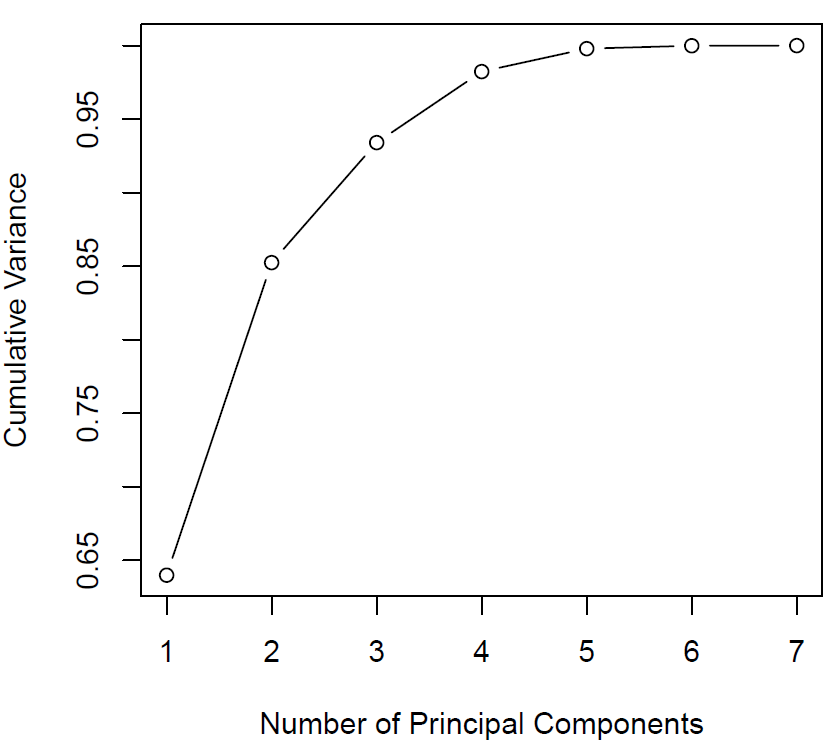


Table S4 Summary OF factor analysis

|  | Factor1 | Factor2 | Factor3 |
| --- | --- | --- | --- |
| Germination Rate | 0.753 | 0.177 | -0.013 |
| Shoot Length | 0.829 | 0.376 | 0.385 |
| Primary Root Length | 0.373 | 0.911 | 0.162 |
| Number of Roots | 0.285 | 0.112 | 0.697 |
| Shoot Fresh Weight | 0.868 | 0.284 | 0.402 |
| Root Fresh Weight | 0.830 | 0.554 | 0.030 |
| Root-to-Shoot Ratio | 0.304 | 0.673 | -0.671 |
